# Supplementary material for: Electrically driven spin resonance of 4f electrons in a single atom on a surface
Source: Nat Commun. 2024 Jun 20;15:5289. doi: 10.1038/s41467-024-49447-y (PMC11190280; doi:10.1038/s41467-024-49447-y)
Supplement: Supplementary file 1 — Supplementary Information [file 41467_2024_49447_MOESM1_ESM.pdf]

Supplementary Information

Electrically Driven Spin Resonance of 4*f* Electrons in a Single Atom on a Surface

Stefano Reale[1,2,3], Jiyoon Hwang[1,4], Jeongmin Oh[1,4], Harald Brune[5], Andreas J. Heinrich[1,4], Fabio Donati[1,4]*, and Yujeong Bae[1,4]*†

[1] Center for Quantum Nanoscience (QNS), Institute for Basic Science (IBS), Seoul 03760, Republic of Korea
[2] Ewha Womans University, Seoul 03760, Republic of Korea
[3] Department of Energy, Politecnico di Milano, Milano 20133, Italy
[4] Department of Physics, Ewha Womans University, Seoul 03760, Republic of Korea
[5] Institute of Physics, Ecole Polytechnique Fédérale de Lausanne, 1015 Lausanne, Switzerland

*Corresponding authors: F.D. (donati.fabio@qns.science), Y.B. (bae.yujeong@qns.science)

†Current address: Empa, Swiss Federal Laboratories for Materials Science and Technology, nanotech@surfaces Laboratory, 8600, Dübendorf, Switzerland

Table of contents

1- **Experimental set-up and identification of atomic species**
2- **Atom manipulation to construct Er-Ti dimers**
3- **Electron spin resonance on isolated Ti and Er**
4- **Effect of the Er-Ti coupling on the ESR spectra of Ti**
5- **Dimer with ^{167}Er**
6- **Tip position dependence of ESR signals on the Er-Ti dimer**
7- **Electron spin resonance spectra with out-of-plane magnetic fields**
8- **Electron spin resonance spectra around the energy level crossing**
9- **Rate equation model**
10- **Measurement of Rabi oscillations**

1- Experimental set-up and identification of atomic species

From the STM image in Fig. S1a, it is possible to distinguish the MgO(100) patch from the Ag(100) substrate by the different apparent height. On top of the MgO patch, different atoms are distinguishable by their distinct apparent heights: ~130 pm for Ti on the oxygen site (Ti_O), ~210 pm for Ti on the bridge site (Ti_B), ~210 pm for Er on the oxygen site (Er_O), ~285 pm for Er on the bridge site (Er_B), and ~170 pm for Fe on the oxygen site as measured at V_{dc} = 100 mV. The species are further identified by their d*I*/d*V* spectra (Fig. S1b–k). While most of the atoms are distinguishable from the apparent heights and the spectral features, the Er_O and Ti_B present a similar apparent height as well as no clear spectral features. To distinguish these two species, we utilize the spin-polarized STM tip. In contrast with Er_O (Fig. S1h), the d*I*/d*V* spectrum measured on Ti_B using the spin-polarized STM tip (Fig. S1g) presents a step-like feature characteristic of a

spin-flip excitation at around zero bias[1] similarly to Ti_O (Fig. S1f). In the main text and in the following sections, we simply refer to Ti_B as Ti and Er_O as Er.

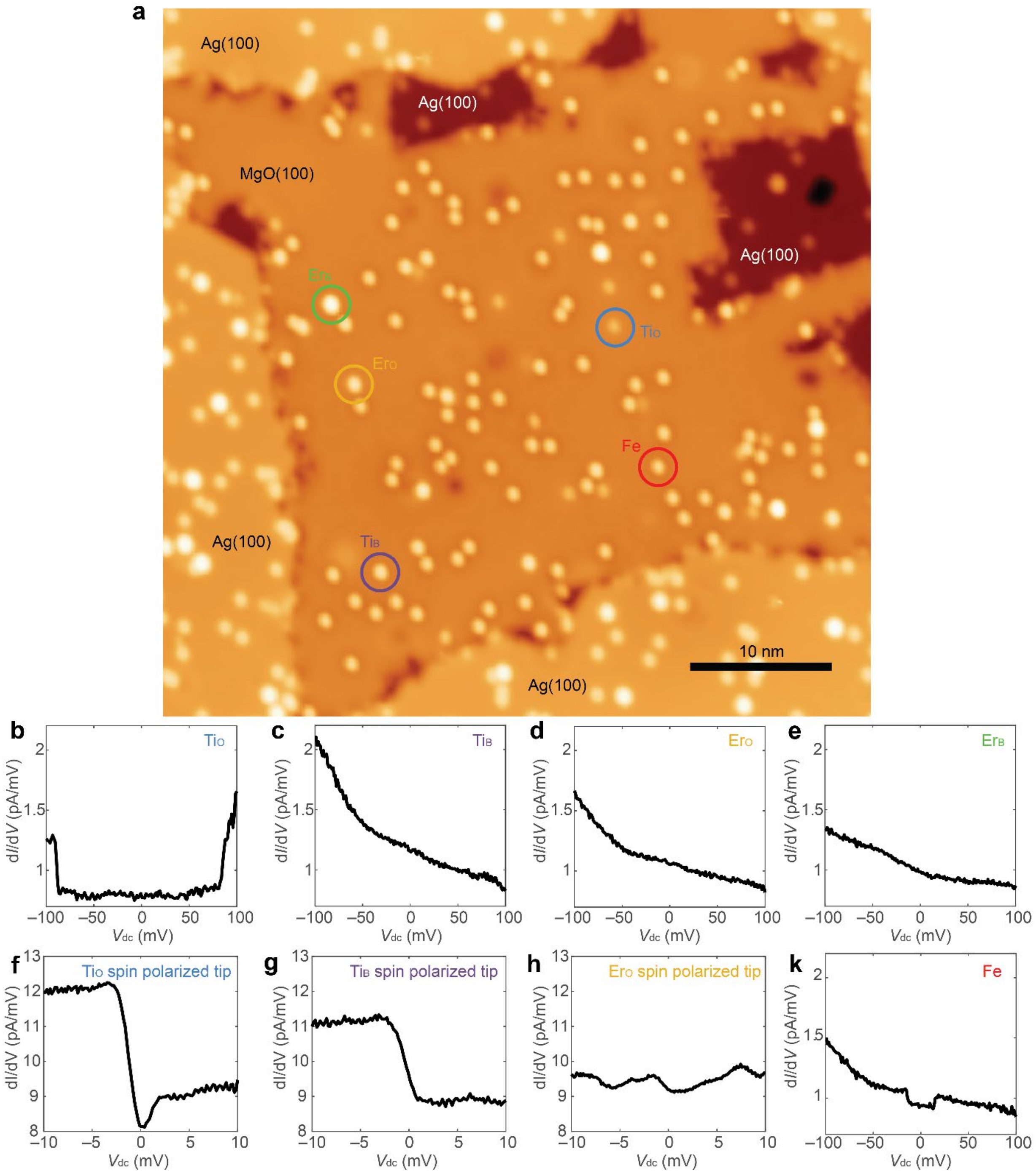

Figure S1 | Characterization of the atomic species. a, Constant current STM image of the Ag(100) surface partially covered by two-monolayers of MgO(100) (set point: V_{dc} = 100 mV, I_{dc} = 20 pA). The different atomic species can be distinguished by their apparent heights and dI/dV features: (**b**) Ti on the oxygen site (Ti_O), (**c**) Ti on the bridge site (Ti_B), (**d**) Er on the oxygen site (Er_O), (**e**) Er on the bridge site (Er_B), and (**k**) Fe. (**f**) Ti_O and (**g**) Ti_B present a spin-flip excitation at around 0 mV when measured with a spin polarized tip, while no excitation is present on Er_O (**h**).

2- Atom manipulation to construct Er-Ti dimers

The Er-Ti dimer used to acquire the data presented in Fig. 1c,d was built through atom manipulation. After identifying a Ti atom and an Er atom, we manipulated the Ti adsorption site by the following procedure: 1) position the STM tip one lattice site away from the Ti center (set point: V_{dc} = 100 mV, I_{dc} = 20 pA), 2) switch off the STM feedback, 3) approach the tip by 330 pm to the surface, 4) apply a voltage pulse of 330 mV, and 5) switch the feedback on. This procedure allows us to move the Ti atom by half lattice sites (from Ti_O to Ti_B and vice versa) in a controlled manner. We repeated this procedure until we obtained the desired Er-Ti dimer with a distance of 0.928 nm (Fig. S2) with the Ti atom placed at the (-2, 2.5) lattice position from Er. We used a similar procedure to preapare the Er-Ti dimers with 0.72 nm separation with the Ti atom placed at the (±2.5, 0) or (0, ±2.5) lattice position from Er.

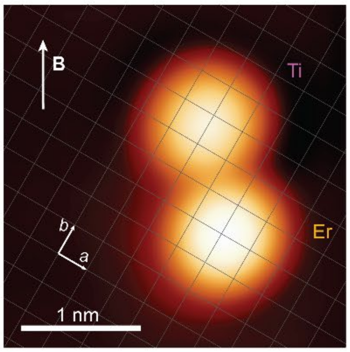

Figure S2 | Constant-current STM image of the Er-Ti dimer with 0.928 nm separation (set point: V_{dc} = 100 mV, I_{dc} = 20 pA). The intersection of grids represents the oxygen site of MgO and the lattice vectors (*a*,*b*) are superimposed on the grid.

3- Electron spin resonance on isolated Ti and Er

In order to perform ESR on an isolated Er atom, we confirmed whether the prepared spin-polarized tip is suitable to perform ESR or not, by measuring the ESR signal on an isolated Ti atom (Fig. S3a). When positioning the same tip over an isolated Er atom, however, no ESR peak was detectable (Fig. S3b).

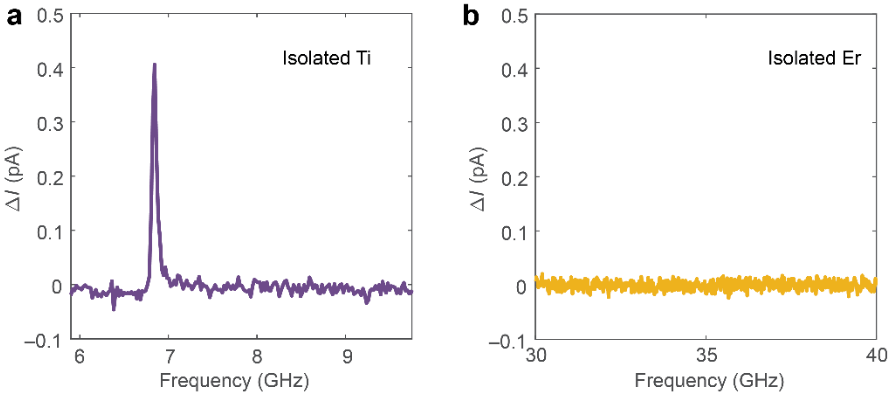

Figure S3 | Electron spin resonances on the isolated atoms. a, ESR spectrum measured with the STM tip positioned above an isolated Ti atom (set point: V_{dc} = 60 mV, I_{dc} = 20 pA, V_{rf} = 20 mV, B = 0.28 T, θ = 97°). **b**, ESR spectrum measured with the STM tip positioned above an isolated Er atom (set point: V_{dc} = 50 mV, I_{dc} = 50 pA, V_{rf} = 20 mV, B = 0.28 T, θ = 97°).

4- Effect of the Er-Ti coupling on the ESR spectra of Ti

To qualitatively explain the effect of the coupling between Er and Ti on the ESR peaks of Ti, we consider both spins as two-level systems. Following Eq. 2 in the main text, we can define the dipolar interaction tensor $\bar{\bar{\mathcal{J}}}_{\mathrm{dip}}$ and the total interaction tensor $\bar{\bar{\mathcal{J}}}_{\mathrm{int}}$ as:

$$\mathbf{J}_{\mathrm{Er}} \cdot \bar{\bar{\mathcal{J}}}_{\mathrm{dip}} \cdot \mathbf{S}_{\mathrm{Ti}} \equiv H_{\mathrm{dip}} \tag{S1}$$

$$\bar{\bar{\mathcal{J}}}_{\mathrm{int}} = \bar{\bar{\mathcal{J}}}_{\mathrm{dip}} + \mathcal{J}_{\mathrm{exc}} \cdot \bar{\bar{\mathbb{1}}}, \tag{S2}$$

where $\mathcal{J}_{\mathrm{exc}}$ is the exchange interaction energy expressed in terms of $\mathbf{J}_{\mathrm{Er}}$ (Eq. 2 in the main text) and $\bar{\bar{\mathbb{1}}}$ is the identity tensor. For a fixed Er-Ti separation at a constant magnetic field angle, the total interaction tensor can be expressed as a scalar $\mathcal{J}_{\mathrm{int}}$. When the magnitude of $\mathcal{J}_{\mathrm{int}}$ is smaller than the Zeeman energy of the two atoms, the ground state of the systems is the $|\downarrow\Downarrow\rangle$ state, where the first and second arrow indicate the Ti and Er spin states, respectively (Fig. S4). At a finite magnetic field and when the interaction is close to zero (Fig. S4a,d), the two resonance frequencies of Ti coincide and only one peak is detected. This case corresponds to the pink spectrum in Fig. 1c in the main text and the spectrum in Fig. S3a. When a detectable interaction is present, the relative energy levels of $|\uparrow\Downarrow\rangle$ and $|\downarrow\Uparrow\rangle$ states shift with respect to the other two states and two peaks become distinguishable in the ESR spectrum. Depending on the sign of $\mathcal{J}_{\mathrm{int}}$, the interaction can be regarded as ferromagnetic (FM) if $\mathcal{J}_{\mathrm{int}} < 0$ (Fig. S4b,e) or antiferromagnetic (AFM) if $\mathcal{J}_{\mathrm{int}} > 0$ (Fig. S4c,f). The transition involving the ground state $|\downarrow\Downarrow\rangle$ (f_1^{Ti}) is characterized by a higher ESR intensity due to its higher population. For this reason, a ferromagnetic interaction, which shifts the antiparallel levels $|\uparrow\Downarrow\rangle$ and $|\downarrow\Uparrow\rangle$ to higher energies, shows the higher intensity peak at higher frequencies. This type of interaction corresponds to the purple spectrum in Fig. 1c in the main text. The opposite is true for an antiferromagnetic interaction which shows the higher intensity peak at lower frequencies. The cases of AFM coupling can be seen in the spectra of the dimer with 0.72 nm separation (Fig. 2b, Fig. 4a in the main text and Fig. S11).

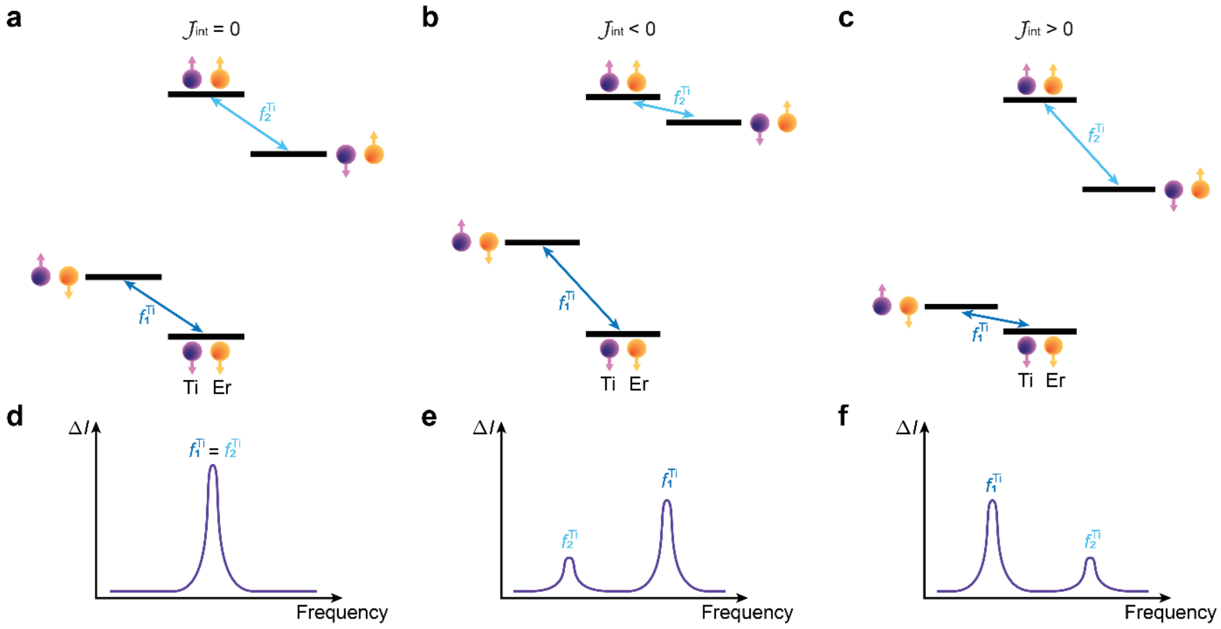

Figure S4 | Influence of the Er-Ti interaction on the energy levels and ESR spectra. a,**b**,**c**, Four-level scheme of Er-Ti dimers with $\mathcal{J}_{\text{int}} = 0$, $\mathcal{J}_{\text{int}} < 0$ (ferromagnetic) and $\mathcal{J}_{\text{int}} > 0$ (antiferromagnetic) respectively. **d**,**e**,**f**, Schematics of the resulting ESR spectra on Ti in its resonance frequency range. When no interaction is present (**a**) only one peak is detectable (**d**). A ferromagnetic interaction ($\mathcal{J}_{\text{int}} < 0$) shifts the antiparallel levels to higher energies (**b**), resulting in two distinguishable peaks with the higher intensity one (f_1^{Ti}) at a higher frequency (**e**). An antiferromagnetic interaction ($\mathcal{J}_{\text{int}} > 0$) shifts the antiparallel levels to lower energies (**c**), resulting in two peaks with the higher intensity one (f_1^{Ti}) at a lower frequency (**f**).

5- Dimer with ^{167}Er

When measuring different Er-Ti dimers at the same separations (0.72 nm), we observed that a small fraction of them do not show any peak in the Er ESR transition range (Fig. S5). We ascribe this observation to the presence of ^{167}Er isotopes on the surface, which is the only observationally stable isotope of Er with a non-zero nuclear spin, present with a 22.9% abundancy. This isotope presents a nuclear spin of 7ħ/2. When driving ESR transitions on this atom, a single ESR peak is expected to split into 8 peaks due to its hyperfine interaction with the nuclear spin[2]. However, as for this atom the intensity of the Er ESR transition is also reduced by a factor of 8 and, thus, the ESR signal becomes too small to be detected in the present detection scheme. Nevertheless, this observation further supports the interpretation that f_3^{Er} and f_4^{Er} correspond to ESR transitions in the Er 4*f* spins since these transitions are the only ones that should be affected by the hyperfine interaction between the Er electron and nuclear spins.

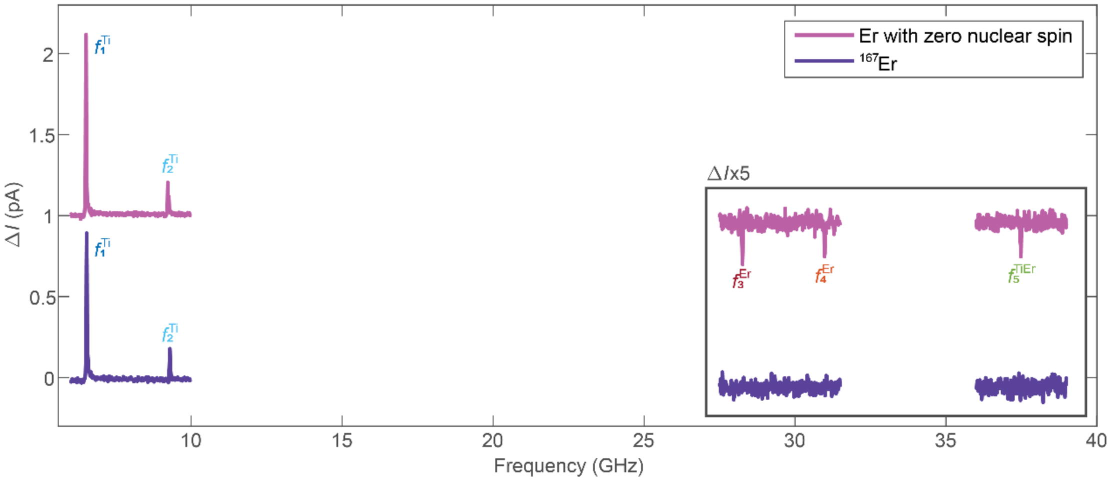

Figure S5 | Electron spin resonance on dimer containing ^{167}Er. ESR spectra acquired with the same STM tip on top of Ti in two different dimers: in the standard Er-Ti dimer (pink line, shifted in Δ*I* by 1 pA for readability) 5 peaks are detectable (f_1^{Ti}, f_2^{Ti}, f_3^{Er}, f_4^{Er} and f_5^{TiEr}) while in the dimer containing ^{167}Er with nuclear spin 7ħ/2 (purple line), only f_1^{Ti} and f_2^{Ti} (related to Ti transitions) are detectable (set point: V_{dc} = 60 mV, I_{dc} = 20 pA, V_{rf} = 15 mV, *B* = 0.3 T, *θ* = 52°).

6- Electron spin resonance spectra with out-of-plane magnetic fields

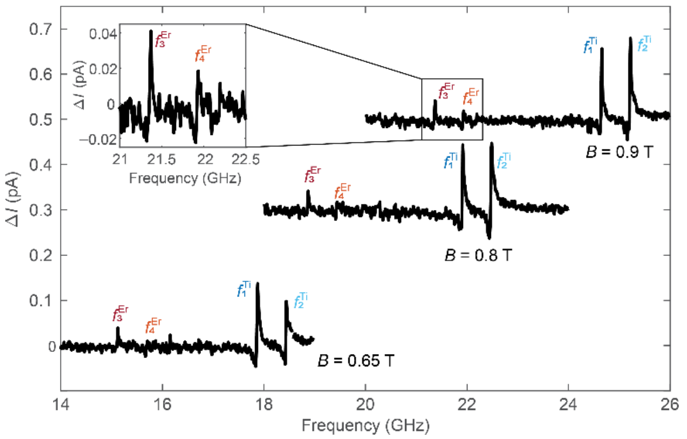

Figure S6 | Electron spin resonance at out-of-plane magnetic fields. ESR spectra measured on the Er-Ti dimer with 0.72 nm separation at different magnetic fields close to the out-of-plane direction (θ = 7°): B = 0.65 T, 0.8 T, and 0.9 T (set point: V_{dc} = 70 mV, I_{dc} = 20 pA, V_{rf} = 25 mV). The spectra at B = 0.8 T and 0.9 T were shifted in ΔI by 0.3 pA and 0.5 pA, respectively, for clarity. The inset on the top left corner shows a zoomed-in spectrum of the peaks f_3^{Er} and f_4^{Er}.

When we applied the magnetic field close to the out-of-plane direction (θ = 7°), we observed 4 ESR peaks (Fig. S6). As explained in the main text, when the magnetic field is applied along the out-of-plane direction (θ = 0°), the expectation value of $\mathbf{J}_{\mathrm{Er}}$ is ħ/2 similarly to $\mathbf{S}_{\mathrm{Ti}}$. However, the Er g-factor is 1.2, while Ti has a g-factor of 1.989 ± 0.024[3]. The peaks at lower frequencies, thus, correspond to the Er ESR transitions (f_3^{Er} and f_4^{Er}) due to the smaller Zeeman energy of Er than the one of Ti. To further clarify the identification of ESR peaks, we measured the ESR speactra at different magnitudes of magnetic fields at θ = 7° and followed the linear dependence of their resonance frequencies on the magnetic field magnitudes.

7- Electron spin resonance spectra close to the level crossing

When the magnetic field is applied at about 12° from the normal to the surface (θ ~12°), we expect Er and Ti to have similar Zeeman splittings. In this situation, the intermediate energy levels of the Er-Ti dimer with 0.72 nm separation must be regarded as singlet $(\frac{1}{\sqrt{2}}|\uparrow\Downarrow\rangle - \frac{1}{\sqrt{2}}|\downarrow\Uparrow\rangle)$ and triplet states[4] $(\frac{1}{\sqrt{2}}|\uparrow\Downarrow\rangle + \frac{1}{\sqrt{2}}|\downarrow\Uparrow\rangle)$ as explained in the main text. In Fig. S7, we show three ESR spectra acquired around the expected matching angle, i.e. θ = 14.5°, 17°, and 22°. At θ = 22°, all four ESR transitions from f_1^{Ti} to f_4^{Er} are visible, with f_3^{Er} and f_4^{Er} observed at higher frequencies than f_1^{Ti} and f_2^{Ti}. In addition, the different peak intensities between f_1^{Ti} and f_2^{Ti} (with the intensity of f_1^{Ti} larger than f_2^{Ti}) indicate an antiferromagnetic coupling between Er and Ti[5]. Conversely, for both θ = 17° and θ = 14.5° , it is not possible to identify the f_3^{Er} and f_4^{Er} peaks stemming from Er ESR transitions. Nevertheless, for both spectra measured for $\theta \leq$ 17° the asymmetric intensity of f_1^{Ti} and f_2^{Ti} is reversed, suggesting a change of the system configuration possibly due to the close match between the Er and Ti levels at around θ = 12°. As discussed in the main text, close to θ = 12° the energy levels of the system cannot be represented as Zeeman product states and for this reason the detection mechanism for both Ti and Er peaks explained in the main text may not be valid in this range of θ.

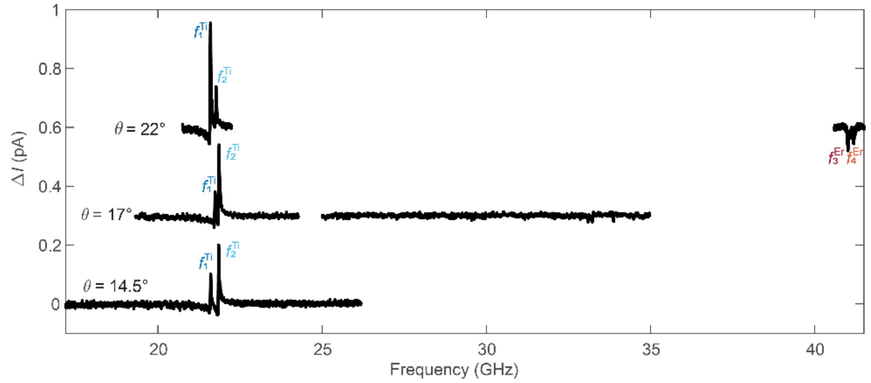

Figure S7 | Electron spin resonance close to the level crossing point. ESR spectra on the Ti atom of the 0.72 nm dimer at θ = 14.5°, 17° and 22° (set point: V_{dc} = 70 mV, I_{dc} = 12 pA, V_{rf} = 20 mV, B = 0.8 T). The spectra at θ = 17° and θ = 22° were shifted in ΔI by 0.3 pA and 0.6 pA, respectively, for clarity.

8- Tip position dependence of ESR signals on the Er-Ti dimer

As mentioned in the main text, when the tip is positioned above the Ti atom in the Er-Ti dimer with 0.72 nm separation, we can resolve up to 5 ESR peaks. However when we move the tip away from the Ti center, the peaks related to the Er ESR transitions decrese in intensity (Fig. S8). When the tip is about 0.3 nm from the Ti center the intensity of the peaks is too low to be resolved (spectrum 2). In a similar way, when the tip is positioned above Er no peaks are detectable (spectrum 1).

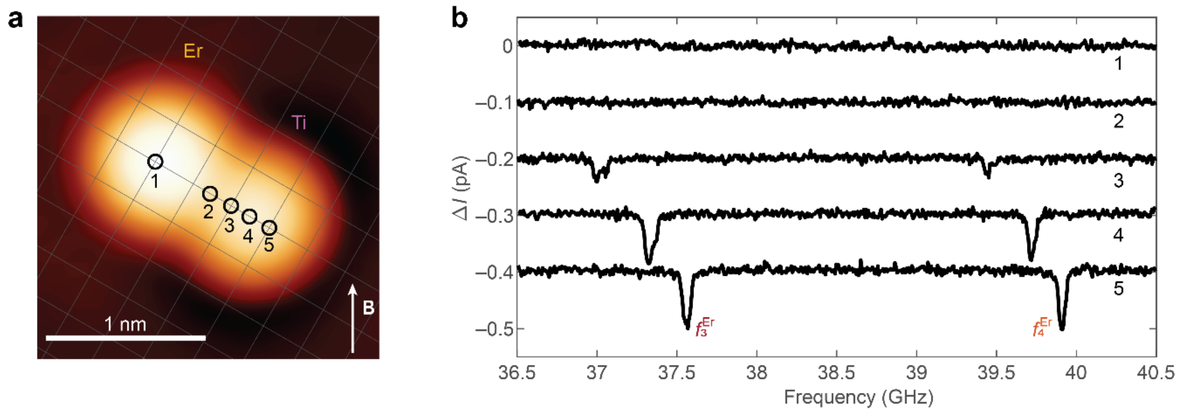

Figure S8 | Electron spin resonance at different tip locations on the Er-Ti dimer. a, STM image of the Er-Ti dimer with 0.72 nm separation (set-point: V_{dc} = 100 mV, I_{dc} = 20 pA) superimposed with a grid representing the oxygen site of MgO lattice. The different locations where the tip was positioned during the ESR measurement are depicted as black circles numbered from 1 to 5. **b**, Corresponding ESR spectra at different tip locations as marked in (**a**) (set-point: V_{dc} = 70 mV, I_{dc} = 12 pA, V_{rf} = 25 mV, B = 0.28 T, θ = 97°). When the tip is approached laterally to the Er atom, the Er ESR peaks, f_3^{Er} and f_4^{Er}, shift to lower frequencies due to the antiferromagnetic interaction between the Er atom and the magnetic tip. The intensity of the peaks decreases with moving the tip away from the Ti atom and no peaks are detectable at a distance of 0.3 nm from its center (spectrum 2). The spectra 2, 3, 4 and 5 were shifted vertically by -0.1 pA, -0.2 pA, -0.3 pA and -0.4 pA, respectively, for clarity.

9- Rate equation model

To reproduce the different sign of the peaks of the Er ESR transitions shown in Fig. 3a, as well as the dependence of the peak intensity as a function of the driving strength (V_{rf}) displayed in Fig. 3b of the main text, we developed a rate equation model based on 4 differential equations:

$$\begin{aligned}
\frac{dn_{00}}{dt} &= -(W_1 + \Gamma_{1-}^{\mathrm{Ti}})n_{00} - (W_3 + \Gamma_{3-}^{\mathrm{Er}})n_{00} + (W_1 + \Gamma_{1+}^{\mathrm{Ti}})n_{10} + (W_3 + \Gamma_{3+}^{\mathrm{Er}})n_{01} \\
\frac{dn_{10}}{dt} &= -(W_1 + \Gamma_{1+}^{\mathrm{Ti}})n_{10} - (W_4 + \Gamma_{4-}^{\mathrm{Er}})n_{10} + (W_1 + \Gamma_{1-}^{\mathrm{Ti}})n_{00} + (W_4 + \Gamma_{4+}^{\mathrm{Er}})n_{11} \\
\frac{dn_{01}}{dt} &= -(W_2 + \Gamma_{2-}^{\mathrm{Ti}})n_{01} - (W_3 + \Gamma_{3+}^{\mathrm{Er}})n_{01} + (W_2 + \Gamma_{2+}^{\mathrm{Ti}})n_{11} + (W_3 + \Gamma_{3-}^{\mathrm{Er}})n_{00} \\
\frac{dn_{11}}{dt} &= -(W_2 + \Gamma_{2+}^{\mathrm{Ti}})n_{11} - (W_4 + \Gamma_{4+}^{\mathrm{Er}})n_{11} + (W_2 + \Gamma_{2-}^{\mathrm{Ti}})n_{01} + (W_4 + \Gamma_{4-}^{\mathrm{Er}})n_{10}
\end{aligned} \qquad \text{(S3)}$$

Here, n_x is the population of the level x, W_y is the driving of the transition y, and Γ_y is the relaxation rate of the levels connected by the transition y. The "+" and "–" subscripts in the relaxation rates indicate if the relaxation is towards a lower energy level (+) or a higher energy level (–), such that the total relaxation can be written as $\Gamma_y = \Gamma_{y+} - \Gamma_{y-}$. We distinguish the total relaxation rates for Er and Ti by computing $\Gamma^{\mathrm{Er}} = 1/T_1^{\mathrm{Er}}$ and $\Gamma^{\mathrm{Ti}} = 1/T_1^{\mathrm{Ti}}$. In addition, we conserve the total population, $n_{00} + n_{10} + n_{01} + n_{11} = 1$. To obtain the steady state solution we set the derivatives equal to zero. By considering a specific driving W_y and solving the set of equations in the steady state, we obtain the population of each level. In Fig. S9a, a schematic of the four-level system is reported with the respective population at thermal equilibrium for each level (the rough numbers for the populations were calculated considering a temperature of 1.3 K and using the energy differences between levels obtained from the ESR peaks of Fig. 2b at B = 0.3 T and θ = 97°). By driving f_3^{Er} into saturation, we can equalize the populations of the $|\downarrow \Downarrow\rangle$ and $|\downarrow \Uparrow\rangle$ states, which makes the system far from the thermal equilibrium state (Fig. S9b). Given that the spin relaxation of Er is much slower than one for Ti, the system tends to relax through a Ti relaxation event (dotted purple arrows in Fig. S9b). The relaxation from $|\downarrow \Uparrow\rangle$ to $|\uparrow \Uparrow\rangle$ will be dominated by upward events while from $|\uparrow \Downarrow\rangle$ to $|\downarrow \Downarrow\rangle$ by downward ones. Finally, by including all the relaxation paths in the model, the population of each level evolves to the steady state reported in Fig. S9c.

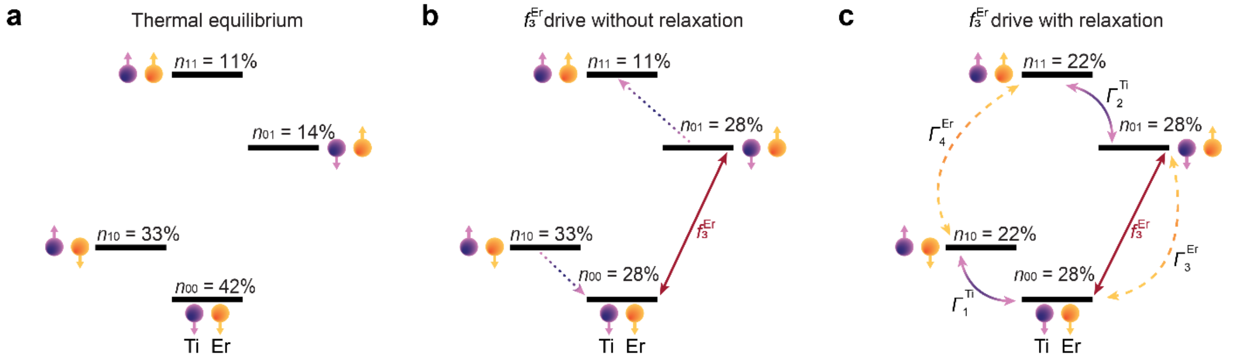

Figure S9 | Populations of the Er-Ti dimer calculated with the rate equation model at different conditions. a, Four-level scheme and respective populations for each level at thermal equilibrium. **b**, Driving the system into resonance at f_3^{Er} (shown as solid red arrow) and prior to activating the relaxation terms described in the model. The ESR driving leads to an equalization of n_{00} and n_{01}, which makes the Ti populations far from the Boltzmann distribution. Dashed arrows represent the relaxation path realized by the Ti relaxation mechanism, where part of the population of n_{10} is transferred to n_{00} through downward relaxation mechanism. The rf excitation re-equilibrates the excess of population towards n_{01}, which is further transferred to n_{11} via upward

Ti relaxation mechanisms. **c**, Resulting populations after the inclusion of the relaxation terms, with the Er relaxation shown as dashed yellow arrows and the Ti relaxation as pink solid arrows.

In the rate equation model, we included the spin pumping term (ζ) as a shift of the population of the levels given by the angular momentum transfer from the tunneling electrons into Ti, as follows:

$$n_{00}^{\text{spin pumping}} = n_{00} - \zeta$$
$$n_{10}^{\text{spin pumping}} = n_{10} + \zeta \qquad \text{(S4)}$$
$$n_{01}^{\text{spin pumping}} = n_{01} - \zeta$$
$$n_{11}^{\text{spin pumping}} = n_{11} + \zeta$$

We used the rate equation model to fit the V_{rf} dependence of the peak intensity in Fig. 3b. We set T_1^{Ti} = 10 ns[6] and T_1^{Er} = 818 ns. We used two different driving terms for Er and Ti transitions: $W^{\text{Ti}} = W_1 = W_2 = A^{\text{Ti}} V_{\text{rf}}^2$ and $W^{\text{Er}} = W_3 = W_4 = A^{\text{Er}} V_{\text{rf}}^2$, where $A^{\text{Ti,Er}}$ is the respective scaling factor for the ESR driving of Ti and Er. The fitting parameters are A^{Ti}, $A^{\text{Er}}$and the spin pumping term ζ. The fitting yielded A^{Ti} = 14917, A^{Er} = 29338 and a negative spin pumping of –0.94%. The driving term can be expressed as $W = T_2 \Omega^2/2$[7]. The rabi rate Ω depends on the strength of the modulation provided by the tip-atom or atom-atom coupling and it is linear with V_{rf} and, thus, the scaling factor A. A four-level scheme depicting all the terms present in the rate equation model is given in Fig. S10a. As discussed in the main text and in the previous section, the Er-Ti coupling is 3–4 times smaller than Fe-Ti dimers used for remote ESR of 3*d* electrons[8,9]. Therefore, we expect a lower Rabi rate for Er since its driving comes from the modulation of its magnetic interaction with Ti. On the other hand, the fit yields a driving factor A^{Er} larger than A^{Ti}, which suggests a much longer T_2 of Er compared to other 3*d* elements, possibly due to the well protected 4*f* orbitals.
The spin pumping term ζ is required to account for the different sign of the Er peaks observed with different ESR tips, as shown in Fig. 3a in the main text. As discussed in the following, our model indicates that a negative ζ produces negative Er peaks while the opposite is true for a positive ζ. The Ti ESR peaks are minimally influenced by ζ. In Fig. S10b, we show the effect of ζ on f_3^{Er} in the rate equation model. In the absence of spin pumping, a small positive signal is predicted. This is due to the difference in energy between the ESR transitions f_1^{Ti} and f_2^{Ti} which leads to an intrinsic difference between Γ_1^{Ti} and Γ_2^{Ti}. Finally, the intensity of the ESR signal is calculated as the difference between the populations of the Ti ↑ states $(n_{10} + n_{11})$ and Ti ↓ states $(n_{00} + n_{01})$ compared to the undriven state:

$$\Delta I = C \cdot \{[(n_{10} + n_{11}) - (n_{00} + n_{01})]_{\text{rf1}} - [(n_{10} + n_{11}) - (n_{00} + n_{01})]_{\text{undriven}}\}, \qquad \text{(S5)}$$

where *C* is a scaling factor which considers the polarization of the tip and the contribution of the magnetic shell on the spin polarized current. To reproduce the experimental results, we use *C* = 45.2 pA. The driven populations are calculated using the differential equations of Eq. S3 and the undriven populations using the ones obtained from Eq. S4.
To reproduce the ESR intensity ratios as a function of V_{rf2} in Fig. 4c, a similar model was employed. With the only difference being the calculation of the ESR intensity as:

$$\Delta I_{\text{double resonance}} = C \cdot \{[(n_{10} + n_{11}) - (n_{00} + n_{01})]_{\text{rf1,rf2}} - [(n_{10} + n_{11}) - (n_{00} + n_{01})]_{\text{rf2}}\} \qquad \text{(S6)}$$

where the driven populations are obtained through Eq. S1 with the application of a simultaneous driving from rf1 and rf2 and subtracted to the population obtained with a single drive rf1 to reproduce the double resonance experiment[9].

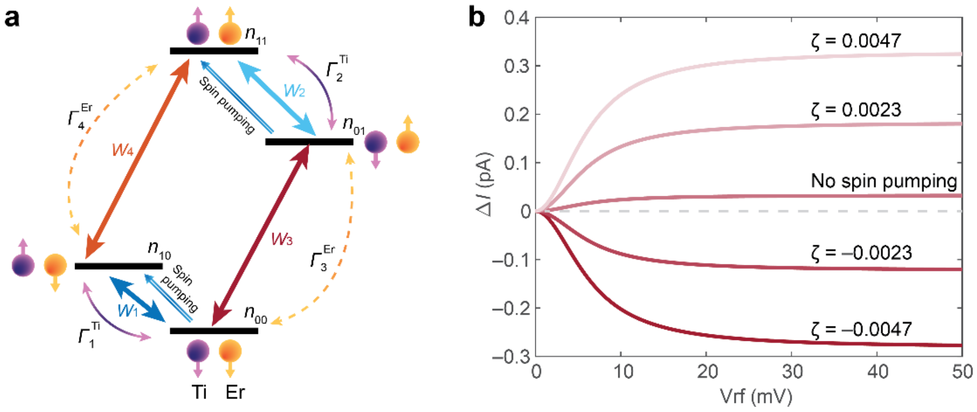

Figure S10 | Rate equation model and spin pumping dependence of the Er ESR signal. a, 4-level scheme relative to the rate equation model describing the driving term (W), relaxation rates (Γ) and a positive spin pumping term (ζ). **b**, Effect of the spin pumping term for the f_3^{Er} peak intensity (ΔI) as a function of V_{rf} predicted by the rate equation model. A negative ζ produces a negative ESR signal while a positive ζ produces a positive ESR signal. When the spin pumping is excluded from the model, a slightly positive ESR signal is predicted.

10- Measurement of Rabi oscillations

To measure Rabi oscillations on Ti and Er atoms in a Er-Ti dimer, we followed a procedure similar to the one used in [6]. With the STM tip positioned on top of the Ti atom in the Er-Ti dimer with 0.72 nm separation, we applied a series of $V_{\rm rf}$ pulses at the resonance frequency of $f_1^{\rm Ti}$ (Fig. a) and $f_3^{\rm Er}$ (Fig. S11b) with increasing pulse widths. We subtracted a linear fit to the data in order to remove the rf rectified current given by the nonlinearity of the *I*-*V* curve[10]. When we apply rf pulses at the resonance frequency of f_1 we can resolve Rabi oscillations in the Ti spin (Fig. S11a). The fit of the signal measured on a Ti atom with an exponentially decaying sinusoidal function yields a Rabi rate $\Omega_{\rm Ti}$ of 435 ± 41 MHz and a $T_{2\,\rm Rabi}^{\rm Ti}$ of 9.9 ± 3.4 ns. On the other hand, when we apply rf pulses at the resonance frequency of f_3, no Rabi oscillation is observed. The monotonic decrease of the signal is due to the negative sign of the f_3 peak, which reaches saturation for sufficiently long rf pulses.

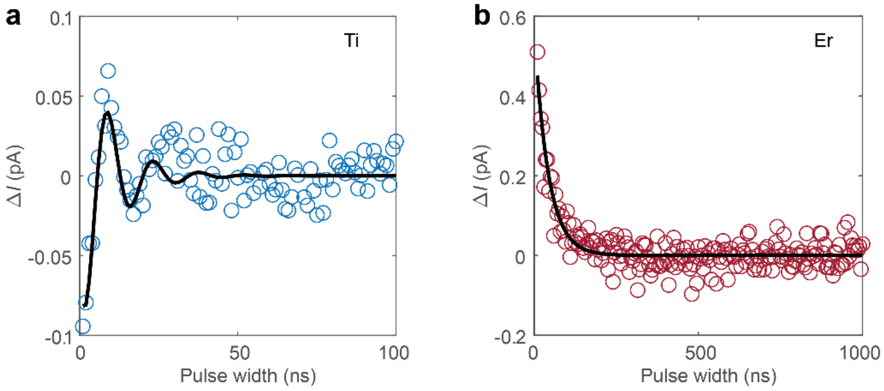

Figure S11 | Rabi measurements. a, Pulsed ESR measured with the STM tip on the Ti atom of the Er-Ti dimer with 0.72 nm separation. With the pulses applied at the resonance frequency of $f_2^{\rm Ti}$, Rabi oscillations for the Ti spin are clearly observed (set-point: $V_{\rm dc}$ = 20 mV, $I_{\rm dc}$ = 10 pA, $V_{\rm rf}$ = 70 mV, B = 0.288 T, θ = 97°). The black line is a fit using an exponentially decaying sine function. **b**, Pulsed ESR measured with the STM tip on the Ti atom of the same dimer but with the pulses at the resonance frequency of $f_3^{\rm Er}$ (set-point: $V_{\rm dc}$ = 70 mV, $I_{\rm dc}$ = 50 pA, $V_{\rm rf}$ = 90 mV, B = 0.288 T, θ = 97°). The black line is an exponential fit as a guide for the eye.

References

1 Loth, S. *et al.* Controlling the state of quantum spins with electric currents. *Nature Physics* **6**, 340-344 (2010). https://doi.org:10.1038/nphys1616

2 Willke, P. *et al.* Hyperfine interaction of individual atoms on a surface. *Science* **362**, 336-339 (2018). https://doi.org:doi:10.1126/science.aat7047

3 Kim, J. *et al.* Spin resonance amplitude and frequency of a single atom on a surface in a vector magnetic field. *Physical Review B* **104**, 174408 (2021). https://doi.org:10.1103/PhysRevB.104.174408

4 Bae, Y. *et al.* Enhanced quantum coherence in exchange coupled spins via singlet-triplet transitions. *Science Advances* **4**, eaau4159 (2018). https://doi.org:doi:10.1126/sciadv.aau4159

5 Yang, K. *et al.* Engineering the Eigenstates of Coupled Spin-1/2 Atoms on a Surface. *Physical Review Letters* **119**, 227206 (2017). https://doi.org:10.1103/PhysRevLett.119.227206

6 Yang, K. *et al.* Coherent spin manipulation of individual atoms on a surface. *Science* **366**, 509-+ (2019). https://doi.org:10.1126/science.aay6779

7 Reiter, F. & Sørensen, A. S. Effective operator formalism for open quantum systems. *Phys. Rev. A* **85**, 032111 (2012). https://doi.org:10.1103/PhysRevA.85.032111

8 Phark, S.-h. *et al.* Electric-Field-Driven Spin Resonance by On-Surface Exchange Coupling to a Single-Atom Magnet. *Advanced Science* **n/a**, 2302033 (2023). https://doi.org:https://doi.org/10.1002/advs.202302033

9 Phark, S.-h. *et al.* Double-Resonance Spectroscopy of Coupled Electron Spins on a Surface. *ACS Nano* **17**, 14144-14151 (2023). https://doi.org:10.1021/acsnano.3c04754

10 Paul, W., Baumann, S., Lutz, C. P. & Heinrich, A. J. Generation of constant-amplitude radio-frequency sweeps at a tunnel junction for spin resonance STM. *Rev. Sci. Instrum.* **87** (2016). https://doi.org:10.1063/1.4955446
